# Immune cell-derived exosomes in the tumor microenvironment: mechanisms of immune evasion and emerging therapeutic strategies in cancer immunotherapy

**DOI:** 10.3389/fimmu.2026.1902856

**Published:** 2026-10-14

**Authors:** Rashid Mir, Mohammad Fahad Ullah, Mamdoh S. Moawadh, Faisal H. Altemani, Jameel Barnawi, Naseh A. Algehainy, Mohammed M. Jalal, Malik A. Altayar, Reema M. Almotairi, Faris J. Tayeb, Ruqaiah I. Bedaiwi, Kholoud S. Almasoudi, Ibrahim Altedlawi Albalawi, Aeshah A. Awaji

**Affiliations:** 1 Prince Fahad Bin Sultan Chair for Biomedical Research, University of Tabuk, Tabuk, Saudi Arabia; 2 Department of Medical Laboratory Technology, Faculty of Applied Medical Sciences, University of Tabuk, Tabuk, Saudi Arabia; 3 Department of Surgical Oncology, Faculty of Medicine, University of Tabuk, Tabuk, Saudi Arabia; 4 Department of Biology, Faculty of Science, University of Tabuk, Tabuk, Saudi Arabia

**Keywords:** tumor-derived exosomes, cancer immunotherapy, extracellular vesicles, immune checkpoint, immunosuppression, myeloid-derived suppressor cells, PD-L1, tumor microenvironment

## Abstract

Tumor-released exosomes have emerged from the shadows of “cellular dust” to be recognized as pivotal mediators of intercellular communication within the tumor microenvironment (TME). These nano-sized vesicles (30–150 nm) carry a complex cargo of proteins, lipids, and nucleic acids that profoundly reshape anti-tumor immunity. This review comprehensively examines the dualistic nature of immune cell-derived exosomes in cancer progression, with particular focus on exosomes originating from B lymphoma cells, T lymphocytes, natural killer (NK) cells, and myeloid-derived suppressor cell lineages including tumor-associated macrophages (TAMs), mast cells, and neutrophils. We delineate the mechanistic pathways through which these exosomes disrupt the cancer immunity cycle suppressing CD8+ T cell activation via PD-L1/PD-1 engagement, inducing lymphocyte apoptosis through FasL and TRAIL, promoting regulatory T cell expansion, polarizing macrophages toward the M2 immunosuppressive phenotype, and establishing pre-metastatic niches through MMP-mediated extracellular matrix remodeling. The molecular machinery governing exosome biogenesis including ESCRT-dependent and ceramide-mediated pathways is critically reviewed, alongside the selective packaging mechanisms that concentrate immunosuppressive miRNAs (miR-155, miR-21, miR-23a, miR-223) into exosomal compartments. Importantly, we highlight the emerging therapeutic premises targeting these vesicles: pharmacological inhibition of exosome secretion (GW4869, Rab27a knockdown), checkpoint blockade reversing exosomal PD-L1-mediated T cell exhaustion, and the engineering of immunostimulatory exosomes as cell-free cancer vaccines establishing exosomes not merely as biomarkers of disease progression but as actionable therapeutic targets. The clinical translation of exosome-based diagnostics including liquid biopsy platforms detecting circulating tumor-derived exosomes and therapeutics that include engineered exosomes delivering immunomodulatory cargo represents a frontier in precision cancer immunotherapy.

## 1 Introduction

Cancer is a major public health problem and the leading cause of death globally, and cancer incidence and mortality are rapidly growing worldwide. By controlling the immune system to reactivate the anti-cancer immune response and block the route leading to tumor escape, cancer immunotherapy is a form of treatment that controls and eliminates tumors (1). Adoptive cell transfer, immune checkpoint blockades, nonspecific immunological activation, and vaccination techniques are the major therapeutic options. The US Food and Drug Administration (FDA) has authorized a number of immunotherapy medications for clinical usage, including cytotoxic T-lymphocyte-associated protein 4 (CTLA-4) inhibitors, programmed cell death 1 (PD-1) inhibitors, and programmed cell death 1 ligand 1 (PD-L1) inhibitors (2). Numerous cell types, including immunological and cancer cells, can produce exosomes, which are single-membrane organelles with a diameter of around 30 to 100 nm (3). Exosomes are mostly composed of cell-derived proteins, lipids, glycoconjugates, and nucleic acids (4). Exosomes perform a number of tasks, including facilitating the intercellular transfer of chemicals and signals and modifying the extracellular matrix (ECM). The dual properties of exosomes in promoting and restraining cancer have been taken into consideration with the investigation of numerous functions of exosomes in the advancement of cancer. Exosomes are cell-derived nanovesicles with potential use in cancer immunotherapy due to their molecular transfer and immunogenicity (5).

Cancer immunotherapy’s potential to boost the immune system, treat a wide range of malignancies, and provide a long-lasting response have made it a research hotspot in recent years. Melanoma, kidney cancer, and non-small cell lung cancer (NSCLC) are only a few of the malignancies in which it has demonstrated potent anti-tumor action (6). In addition to activating autocrine VEGF signaling in endothelial cells to enhance tumor angiogenesis, exosomes produced by cancer cells can modify many stromal cell types to support cancer cell proliferation and invasive behavior (7). Exosomes produced from cancer can depress the immune system by preventing CD8+ T cell activation and proliferation and encouraging the growth of regulatory T cells (8). Furthermore, exosomes have lately been shown to have some unexpected anti-cancer properties. Numerous major histocompatibility complex class I molecules (MHC I) and tumor markers, such as heat shock proteins (HSP), which are involved in antigen presentation and T cell stimulation and have been demonstrated to initiate CD8+ T cell-dependent anti-tumor responses *in vitro* and *in vivo*, have been found to be expressed by dendritic cell (DC)- and tumor-derived exosomes (9). Therefore, how exosomes may be used in immune treatment is crucial in terms of cancer progression and has implications for diagnostics and the development of innovative therapeutic techniques since they are carriers to trigger anti-cancer immune responses and deliver anti-cancer medications. The purpose and mechanism of exosome-based immunotherapy in human cancer, its important therapeutic impact on cancer progression, and the potential for creating immunotherapeutic vaccines were the main topics of this study.

## 2 Tumor-released exosomes

Exosomes are nano-sized extracellular vesicles (30–150 nm) secreted by virtually all cell types through the endosomal pathway. Within the tumor microenvironment (TME), both malignant and immune cells exploit these vesicles as intercellular messengers, transferring bioactive cargo — including proteins, lipids, mRNAs, and non-coding RNAs — that profoundly shapes immune responses (10, 11). Tumor-released exosomes are particularly adept at reprogramming recipient immune cells to foster a permissive environment for tumor growth and immune escape. To appreciate how exosomal immunosuppression operates, it is first important to understand the cancer immunity cycle — the multistep process through which the immune system detects and destroys cancer cells (**Figure 1**) — as tumor-released exosomes disrupt this cycle at multiple interconnected steps (13, 14).

**Figure 1:**
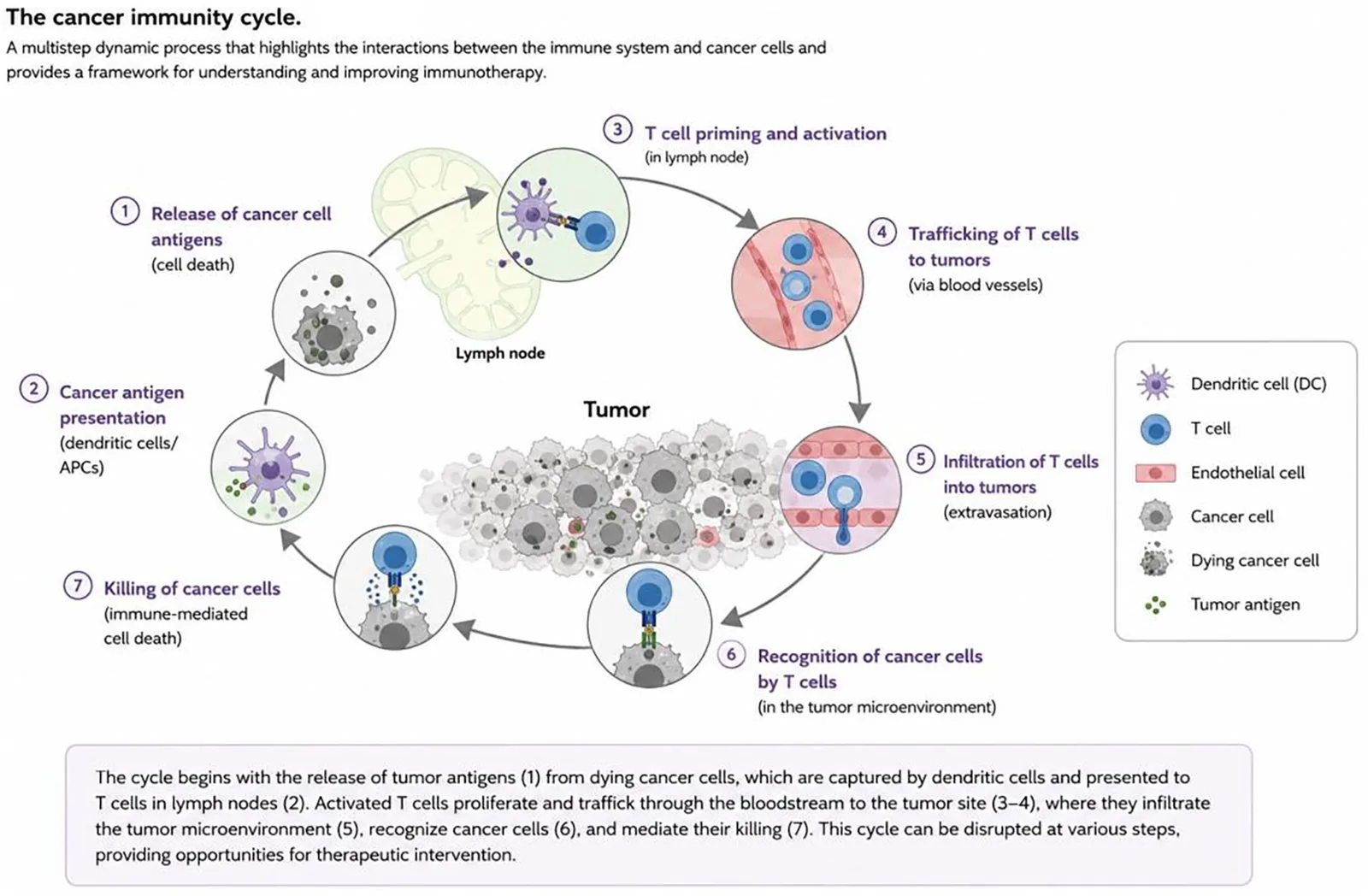
The cancer immunity cycle. A multistep dynamic process that highlights the interactions between the immune system and cancer cells. The cycle begins with release of tumor antigens (step 1) from dying cancer cells, captured by dendritic cells and presented to T cells in lymph nodes (step 2). Activated T cells traffic through the bloodstream (steps 3–4), infiltrate the tumor microenvironment (step 5), recognize cancer cells (step 6), and mediate immune-mediated killing (step 7). Tumor-released exosomes from B lymphoma cells, T lymphocytes, and NK cells disrupt this cycle predominantly at steps 2, 3, 6, and 7, diverting the immune response toward tumor tolerance. Adapted from Chen & Mellman (12).

The biogenesis of immunosuppressive exosomes within tumor-associated immune cells is a tightly regulated process governed by endosomal sorting complexes required for transport (ESCRT) machinery and ceramide-dependent pathways. Upon endocytosis, early endosomes mature into late endosomes also termed multivesicular bodies (MVBs) — through the inward budding of intraluminal vesicles (ILVs). The molecular sorting of immunosuppressive cargo into these ILVs is not random: specific RNA-binding proteins (hnRNPA2B1, YBX1) and lipid raft-associated proteins selectively package miRNAs such as miR-21 and miR-155 into ILVs destined for secretion, while excluding pro-inflammatory cargo from the exosomal compartment (11). MVBs then either fuse with lysosomes for degradation or dock with the plasma membrane via Rab27a/b-dependent vesicular trafficking, releasing ILVs as exosomes into the extracellular space. The balance between these two fates — degradation versus secretion — is critically regulated by the immunological activation state of the cell: immunosuppressive signals (IL-10, TGF-β) promote Rab27a expression and exosome secretion, while pro-inflammatory stimuli (IFN-γ, LPS) divert MVBs toward lysosomal destruction. This mechanistic foundation explains why tumor-educated immune cells secrete a dramatically altered exosomal output compared with their non-conditioned counterparts (10, 15).

A pattern recurs across the immune lineages considered below — B lymphoma cells, T lymphocytes, and NK cells: exosomes secreted by these otherwise cytotoxic or antigen-presenting effectors are progressively repurposed, under sustained TME conditioning, into vehicles that silence the very functions their parent cells normally perform. Rather than simply mirroring parental activation state, immune cell-derived exosomes appear to actively propagate and amplify TME-driven suppression — converting each lineage’s own cytotoxic or immunostimulatory cargo into an instrument of its functional silencing and that of neighboring effector cells. This framing is offered as an organizing lens for the subsections that follow rather than as an established, uniformly demonstrated mechanism; the strength of evidence for it varies considerably by cell type and is noted where relevant below.

### 2.1 B lymphoma cell-derived exosomes

Healthy B lymphocytes rely on antibody production and antigen presentation to support anti-tumor immunity, and their exosomes physiologically contribute to this role by carrying MHC-peptide complexes and co-stimulatory molecules that help activate T cells. In B cell lymphomas — a heterogeneous group of hematological malignancies arising from B lymphocytes at various stages of differentiation — this same secretory pathway is redirected: neoplastic B cells secrete exosomes carrying immunosuppressive cargo capable of subverting the anti-tumor immunity their non-malignant counterparts would normally support (16, 17). *In vitro* and murine model data indicate that these exosomes are enriched in programmed death-ligand 1 (PD-L1), a critical immune checkpoint molecule that, upon docking with PD-1 receptors on cytotoxic T lymphocytes (CTLs), induces T cell exhaustion and functional silencing (18); direct confirmation of the magnitude of this effect in primary patient-derived lymphoma exosomes would strengthen the claim. The molecular mechanism by which PD-L1 and CTLA-4 checkpoint signaling suppresses T cell function — and how this can be reversed by checkpoint blockade antibodies — is illustrated in **Figure 2**.

**Figure 2:**
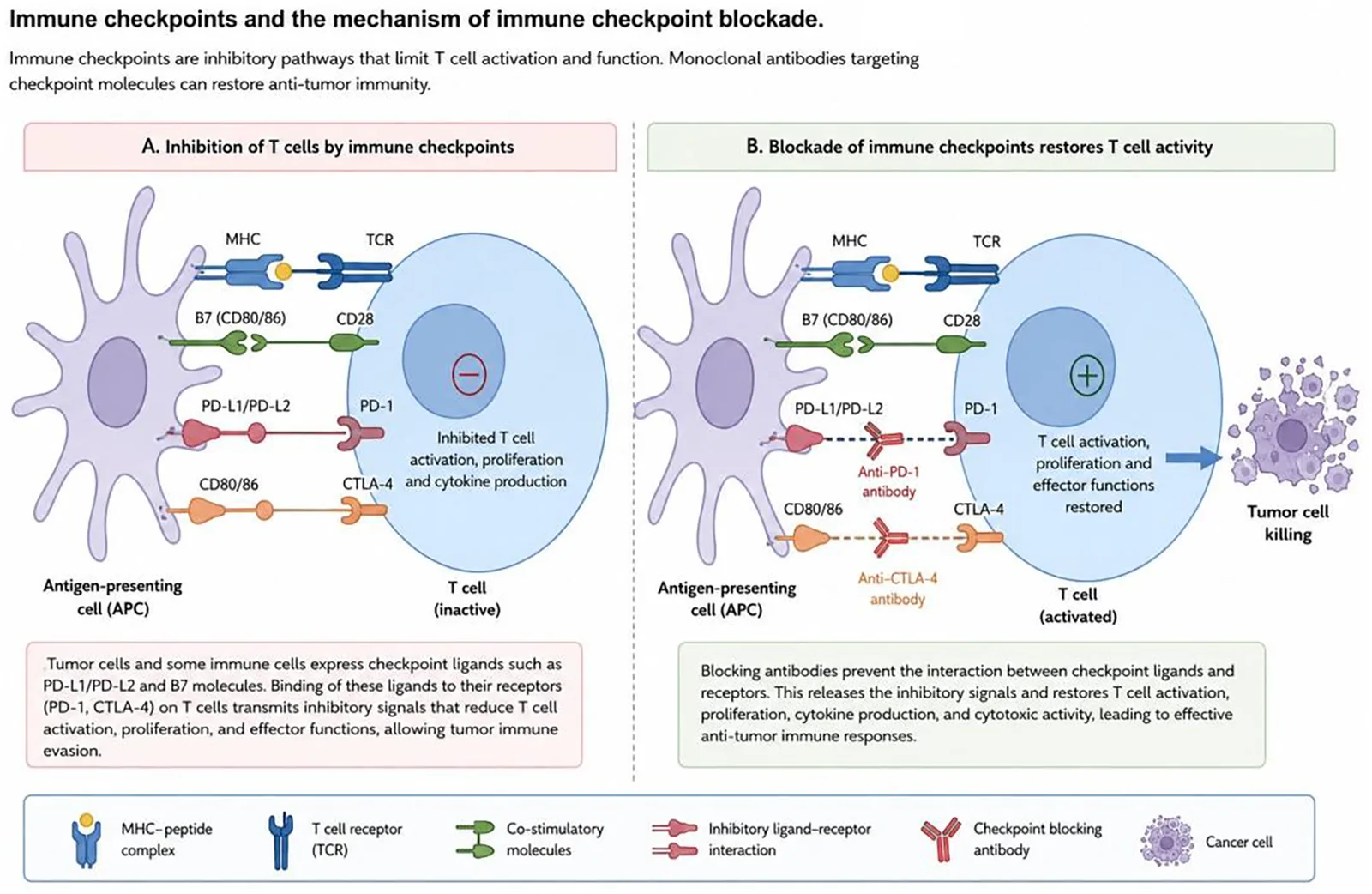
Immune checkpoints and the mechanism of immune checkpoint blockade. **(A)** Tumor cells and exosomes from B lymphoma cells express checkpoint ligands including PD-L1/PD-L2, which bind PD-1 on T cells to transmit inhibitory signals that suppress T cell activation, proliferation, and effector functions. CTLA-4 engagement with CD80/86 provides a parallel inhibitory signal. **(B)** Monoclonal antibodies targeting PD-1 (e.g., pembrolizumab) or CTLA-4 (e.g., ipilimumab) block these interactions, restoring T cell activation and cytotoxic anti-tumor responses. B lymphoma-derived exosomal PD-L1 mimics tumor cell checkpoint signaling at a distance, representing a non-contact mechanism of immune evasion. Adapted from checkpoint biology concepts reviewed in Pardoll (19).

B-lymphoma-derived exosomes (BLDEs) package a distinct repertoire of microRNAs (miRNAs), including miR-155, miR-21, and miR-146a, which have been implicated in the suppression of innate and adaptive immune signaling pathways in dendritic cells (DCs) and macrophages (20). miR-155 is a well-characterized negative regulator of SHIP1, an inositol phosphatase that normally constrains inflammatory signaling; in myeloid cells this can paradoxically dampen TLR-driven inflammatory maturation, and an analogous effect on DC maturation and subsequent CTL priming within the TME has been proposed for BLDE-delivered miR-155, although direct experimental confirmation in the B-lymphoma-exosome setting remains limited and warrants dedicated validation. Moreover, BLDEs carry Fas ligand (FasL) on their surface, inducing apoptosis in activated CD8+ T cells that have infiltrated the tumor vicinity, decimating the cytotoxic arm of adaptive immunity (21).

Mechanistically, BLDEs are taken up by recipient cells through macropinocytosis and clathrin-mediated endocytosis, with cellular tropism determined partly by surface tetraspanin expression patterns (CD9, CD63, CD81), based on *in vitro* uptake studies (22). BLDEs have also been detected in the peripheral blood of diffuse large B-cell lymphoma (DLBCL) patients, using plasma exosome isolation protocols validated for cancer patient samples (23); whether their concentration correlates with disease stage and treatment resistance, as has been suggested, requires confirmation in a dedicated DLBCL biomarker study, but the underlying detectability supports their potential as liquid biopsy candidates.

The immunosuppressive influence of BLDEs extends beyond direct CTL silencing. These vesicles facilitate Treg expansion by delivering TGF-β1 and IL-10 to the TME (24), and have been proposed to polarize TAMs toward the M2 immunosuppressive phenotype through coordinated exosomal delivery of IL-10 and miR-21, drawing on an analogous M2-polarizing mechanism demonstrated for exosomes from other tumor types such as glioblastoma stem cells (25); direct confirmation of this pathway for BLDEs specifically has not yet been reported. Proteomic profiling of BLDEs has additionally revealed surface expression of galectin-9, which engages TIM-3 on NK cells and CTLs to drive receptor-mediated exhaustion (26). Clinically, elevated circulating BLDE-associated PD-L1 has been proposed as a marker of inferior progression-free survival in DLBCL and follicular lymphoma patients, though this specific prognostic association requires direct confirmation in the primary DLBCL/follicular-lymphoma literature; pharmacological inhibition of nSMase2-dependent exosome secretion using GW4869, shown to reduce exosomal microRNA release in other tumor contexts (27), is a candidate strategy for restoring CTL-mediated tumor killing that warrants B-lymphoma-specific preclinical testing.

From a translational perspective, BLDEs represent a dual opportunity: as therapeutic targets and as non-invasive disease biomarkers. The surface proteome of BLDEs — enriched in PD-L1, galectin-9, FasL, and tetraspanins — is sufficiently distinct from healthy B cell-derived exosomes to allow discrimination by multi-parameter flow cytometry or nanoparticle tracking analysis, opening the possibility of liquid biopsy-based disease monitoring without the need for bone marrow biopsy (23). Additionally, the miRNA cargo of BLDEs (particularly miR-155 and miR-21 signatures) has been proposed as a complement to conventional lactate dehydrogenase and beta-2 microglobulin biomarkers for monitoring treatment response in DLBCL patients undergoing R-CHOP chemoimmunotherapy. As our mechanistic understanding of BLDE biogenesis deepens, rational combination strategies — pairing exosome secretion inhibitors with anti-PD-1 checkpoint blockade — are likely to emerge as clinically actionable approaches in relapsed/refractory B cell lymphoma (15).

### 2.2 T lymphocyte cell-derived exosomes

Unlike B lymphoma cells or the myeloid populations discussed later, T lymphocytes do not have a single dominant exosomal phenotype: their immunological consequences are critically dependent on the originating T cell subset and the prevailing cytokine milieu, so the same lineage can yield either suppressive or cytotoxic exosomal output depending on context. Regulatory T cell-derived exosomes (Treg-exos) illustrate the suppressive end of this spectrum, carrying high surface concentrations of TGF-β1, CTLA-4, and neuropilin-1 that directly inhibit CD8+ effector T cell proliferation and cytotoxic function in co-culture assays (28–30). Treg-exos have additionally been reported to carry immunoinhibitory surface ligands capable of extending suppressive signaling to bystander NK cells within the TME, although the specific molecular cargo responsible for this NK-directed effect remains to be fully characterized (31). CD39 and CD73 ectoenzymes on Treg-exo surfaces convert extracellular ATP into immunosuppressive adenosine, impairing CTL function through A2A receptor-mediated cAMP accumulation *in vitro* (31, 32).

Exosomes from activated CD8+ CTLs carry perforin, granzyme B, and FasL, reflecting the cytotoxic cargo of the lysosome-related effector vesicles from which they derive, and have demonstrated anti-tumor activity *in vitro* (33). However, prolonged TME-driven antigen stimulation is thought to generate exhausted T cell-derived exosomes that lose cytotoxic cargo and instead display inhibitory receptors such as LAG-3 and TIM-3, potentially propagating bystander T cell exhaustion; this specific exosome-mediated mechanism remains preliminary and requires direct confirmation in dedicated primary studies.CD4+ Th1-derived exosomes (miR-29a-enriched) promote M1 macrophage polarization, while Th2-derived exosomes (IL-4, IL-13) shift macrophages toward immunosuppressive M2 phenotypes (34, 35). Therapeutic reprogramming of T cell-derived exosomes — engineering them with co-stimulatory ligands or immune-stimulatory cargo — represents a compelling cell-free immunotherapy frontier (5).

The metabolic context of T cell activation critically shapes the composition of T cell-derived exosomes. Under hypoxic TME conditions, HIF-1α stabilization in T cells promotes preferential sorting of immunosuppressive cargo — including PD-L1 and miR-210 — into exosomes, while reducing the packaging of cytotoxic granzyme B. This hypoxia-driven remodeling of the T cell exosomal proteome creates a vicious cycle in which poorly vascularized tumors generate a local exosomal environment that further suppresses infiltrating CTLs (32).Moreover, nutrient deprivation — particularly glucose and arginine depletion — within the TME activates AMPK signaling in T cells, which has been shown, *in vitro* under nutrient-restricted culture conditions, to alter exosome biogenesis by shifting MVB fate from the secretory pathway toward lysosomal degradation, effectively reducing total exosome output from effector T cells while Treg exosome secretion remains relatively preserved (31).This differential metabolic sensitivity means that the TME metabolic milieu systematically tilts the balance of T cell-derived exosomes toward immunosuppression, reinforcing the need to combine metabolic reprogramming strategies with exosome-targeted therapies.

Taken together, the T lymphocyte exosome compartment illustrates how a single cell lineage can generate opposing immunological signals depending on subset identity and TME conditioning — a duality that complicates simple therapeutic targeting but also opens subset-specific intervention points for future immunotherapy design.

### 2.3 Natural killer cell-derived exosomes

Of the three lymphoid populations considered in this section, NK cells arguably show the clearest example of TME-driven reversal: they begin as innate lymphoid cells whose defining function is rapid, MHC-unrestricted cytotoxicity against transformed cells (36), and their exosomes initially reflect that function — NK-exos from non-activated or ex vivo-expanded NK cells are enriched in perforin and granzyme B, demonstrating significant anti-tumor activity *in vitro* (37, 38). Within the immunosuppressive TME, however, this cytotoxic exosomal output is progressively undermined by two distinguishable routes. First, NK cell function is impaired indirectly: hypoxic tumor-derived microvesicles transfer TGF-β1 and miR-23a to NK cells, downregulating the activating receptor NKG2D and the degranulation marker CD107a/LAMP1 (39). Second, and distinct from this tumor-to-NK-cell route, tumor-conditioned NK-exos have also been proposed to transfer inhibitory receptors such as KIR2DL1 and NKG2A to neighboring NK cells, further impairing cytolytic capacity, although this specific inhibitory-receptor-transfer mechanism requires confirmation in a dedicated primary study rather than extrapolation from NK-exo literature describing therapeutic or antitumor effects.

Under physiological conditions, NK-exos stimulate DC maturation and cross-presentation of tumor antigens, amplifying adaptive anti-tumor immunity (40).Within the TME, immunosuppressed NK-exos have been proposed to deliver miR-let-7b and miR-146a to DCs, suppressing TLR signaling and IL-12 secretion and dismantling the NK–DC immunological axis; this specific suppressive mechanism draws on NK-exo cargo-transfer literature that otherwise emphasizes augmentation of antitumor NK activity (41), so the immunosuppressive framing here should be treated as a proposed extension rather than a directly demonstrated finding. Therapeutically, in preclinical tumor models, ex vivo NK-exos decorated with anti-EpCAM ligands and loaded with immune-stimulatory miRNAs show synergistic tumor-killing combined with anti-PD-1 blockade (42), and, *in vitro*, TRAIL-displaying NK-exos overcome apoptosis resistance in TRAIL-refractory tumor cells (43). Collectively, NK cell-derived exosomes illustrate a critical duality: cytotoxic effectors in normal physiology, vehicles of immune dysfunction once TME-conditioned. Preserving or restoring the cytotoxic NK-exo phenotype while blocking inhibitory receptor transfer represents a high-priority avenue in NK cell-based cancer immunotherapy.

The molecular switches governing the transition from cytotoxic to immunosuppressive NK-exo phenotypes remain incompletely defined, but TGF-β signaling is a leading candidate node. Sustained TGF-β1 exposure downregulates the activating receptors NKp30 and NKG2D on NK cells (44), and, as noted above, TGF-β1 co-delivered with miR-23a via hypoxic tumor-derived microvesicles further suppresses NK effector function (39) — together pointing to TGF-β as a convergent immunosuppressive signal rather than an NK-cell-intrinsic biogenesis switch for miR-23a, a distinction that has not always been made clearly in the exosome literature. Blocking TGF-β signaling — either with small molecule inhibitors (e.g., galunisertib) or anti-TGF-β neutralizing antibodies — has been shown to partially restore NK cytotoxic function in ex vivo culture systems, suggesting that systemic TGF-β blockade may concomitantly reinvigorate NK cell exosomal cytotoxicity alongside its established effects on direct NK–tumor cell contact (44).These findings reinforce the concept that the NK-exo phenotype is not hardwired but is dynamically regulated by the TME, and that pharmacological intervention at the level of NK cell conditioning can reshape the immunological output of their exosomal secretome. These lymphoid-lineage mechanisms are summarized in **Table 1**.

**Table 1:** Tumor-released immune cell-derived exosomes: cell source, key molecular cargo, immunosuppressive mechanisms, and cited references.

Cell source	Key molecular cargo	Primary immunosuppressive mechanism	Key references
B lymphoma	PD-L1, FasL, miR-155, miR-21, miR-146a, TGF-β1, IL-10, galectin-9	CTL exhaustion via PD-1/PD-L1; CTL apoptosis via FasL; DC dysfunction via miR-155; Treg expansion; M2 TAM polarization; NK cell exhaustion via TIM-3	16, 18
T lymphocyte (Treg-exos)	TGF-β1, CTLA-4, neuropilin-1, miR-23a, CD39, CD73	CD8+ T cell inhibition; NK cell degranulation blockade via LAMP1 silencing; adenosine-mediated CTL suppression	15, 30
T lymphocyte (exhausted CTL-exos)	LAG-3, TIM-3 ectodomains	Bystander T cell exhaustion spreading; loss of perforin/granzyme B cytotoxic cargo	45 (mechanism preliminary; see main text)
Natural killer	miR-23a, miR-let-7b, miR-146a, KIR2DL1, NKG2A (non-activated: perforin, granzyme B)	NK cell dysfunction via LAMP1 silencing; DC IL-12 suppression; inhibitory receptor transfer to bystander NK cells	38, 46

Beyond the lymphoid compartment, myeloid cells — tumor-associated macrophages (TAMs), mast cells, and neutrophils — represent a mechanistically distinct arm of exosome-mediated immunosuppression within the tumor microenvironment (TME). Unlike the lymphoid populations described above, these three myeloid populations do not share a common suppressive lineage: each differentiates along its own developmental pathway and is independently reprogrammed toward a pro-tumor phenotype by TME-derived signals. Exosomes secreted by TAMs, mast cells, and neutrophils nonetheless extend this reprogramming beyond the cells’ immediate anatomical location, further entrenching immune evasion while simultaneously driving angiogenesis, metastasis, and therapy resistance.

## 3 Myeloid cell-derived exosomes: TAM, mast cell, and neutrophil lineages

It is important to clarify at the outset that tumor-associated macrophages (TAMs), mast cells, and neutrophils are distinct, terminally differentiating myeloid populations and are not themselves classified as myeloid-derived suppressor cells (MDSCs). MDSCs are a separate, heterogeneous population of pathologically activated immature myeloid cells — broadly subdivided into monocytic MDSCs (M-MDSCs, CD14+CD11b+HLA-DRlo/−) and polymorphonuclear MDSCs (PMN-MDSCs, CD15+CD11b+) — that expand dramatically in response to tumor-derived signals including G-CSF, VEGF, and IL-6, and that suppress anti-tumor immunity through ROS production, L-arginine depletion via Arg-1, and secretion of immunosuppressive cytokines (47–50). TAMs, mast cells, and neutrophils share several of these effector mechanisms, and in some tumor contexts can arise from overlapping myeloid precursor pools, but they remain functionally and developmentally distinct populations that are best considered separately rather than grouped under the MDSC umbrella. The subsections below examine how exosomes secreted by each of these three myeloid populations independently extend immunosuppression well beyond their anatomical location (31, 51). These myeloid-targeted therapeutic strategies are summarized in **Figure 3**.

**Figure 3:**
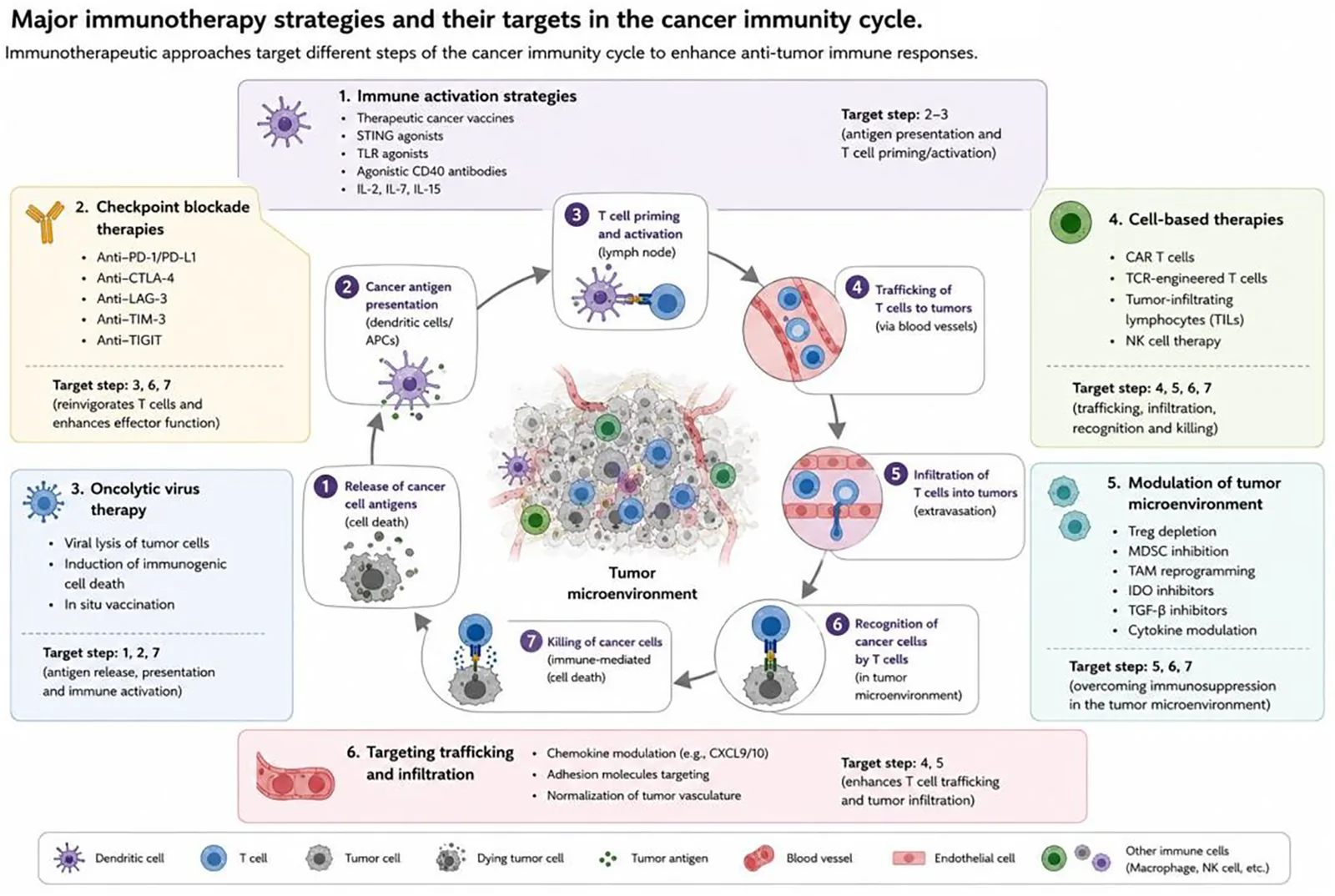
Major immunotherapy strategies and their targets in the cancer immunity cycle, contextualizing the therapeutic relevance of TAM-, mast cell-, and neutrophil-derived exosome biology. Strategy 5 (Modulation of tumor microenvironment) - encompassing Treg depletion, MDSC inhibition, TAM reprogramming, IDO inhibitors, TGF-β inhibitors, and cytokine modulation — directly addresses the mechanisms driven by TAM-derived, mast cell-derived, and neutrophil-derived exosomes described in this section. TAM-exo biogenesis inhibition (GW4869, Rab27a knockdown) maps to TAM reprogramming (target steps 5, 6, 7). Mast cell stabilizers and VEGFR2 inhibition map to cytokine modulation and tumor vascularization targeting. PAD4 inhibition and miR-223 antagomirs for neutrophil NDE suppression map to myeloid-targeted inhibition strategies within Strategy 5. Adapted from the cancer-immunity-cycle framework of Chen and Mellman (12).

A critical but underappreciated feature of these myeloid-derived exosomes is their capacity to engage in bidirectional cross-talk not only with tumor cells and lymphocytes but also among myeloid cell populations themselves. In preclinical models, TAM-exos have been shown to promote the differentiation of circulating monocytes into additional immunosuppressive M2 macrophages upon arrival at the tumor site, effectively pre-conditioning incoming myeloid cells before they encounter tumor-derived polarization signals (52). Similarly, *in vitro*, mast cell-derived exosomes can activate resting neutrophils toward an N2-like pro-tumor phenotype through exosomal IL-13 and PGE2 delivery, creating a cooperative immunosuppressive network between mast cells and neutrophils within the TME (53, 54). This inter-myeloid exosomal signaling is particularly significant because it means that targeting any single myeloid population with exosome inhibitors may be insufficient — the remaining populations can partially compensate through exosome-mediated reprogramming of recruited monocytes and neutrophils. These findings argue strongly for combination approaches that simultaneously target TAM, mast cell, and neutrophil exosome secretion pathways, or that block shared downstream effectors such as TGF-β1, IL-10, and PD-L1 at the recipient cell level (31, 50).

### 3.1 Tumor-associated macrophage-derived exosomes

Macrophages are highly plastic cells capable of both pro-inflammatory (M1) anti-tumor and immunosuppressive (M2) pro-tumor states, and it is this plasticity — rather than a fixed identity — that tumor-associated macrophages (TAMs) exploit within the TME. TAMs are among the most abundant immune cells in many solid tumors and predominantly settle into an M2-like immunosuppressive phenotype under the influence of IL-4, IL-13, IL-10, and TGF-β (52), though the same cells retain the capacity to be repolarized toward an M1, antitumor state under appropriate therapeutic pressure (a duality explored further below). TAM-derived exosomes (TAM-exos) carry cargo enriched in anti-inflammatory cytokines, oncogenic miRNAs, and immune checkpoint molecules, and have been proposed, largely on the basis of *in vitro* co-culture and murine tumor models, to deliver IL-10 and TGF-β1 to CD8+ T cells that suppress TCR signaling and upregulate inhibitory receptors (PD-1, LAG-3, TIM-3), collectively driving T cell exhaustion (55); this specific TAM-exo-to-CD8+-T-cell mechanism draws on the broader tumor-exosome literature and would benefit from TAM-exo-specific confirmation, ideally in primary human samples. *In vitro*, exosomal miR-21 has similarly been implicated in silencing PTEN in CD4+ T cells, activating PI3K/AKT and promoting Treg differentiation (20, 56).

Beyond immune suppression, TAM-exos have been reported to deliver VEGF-A and miR-223 to endothelial cells, stimulating angiogenic sprouting and vascular maturation; this parallels an analogous VEGF-exosome transfer mechanism well established for other stromal cell exosomes in the TME, such as cancer-associated fibroblast-derived exosomes (57), though TAM-exo-specific confirmation of this exact pathway is still warranted. TAM-exos have also been reported to carry EMT-promoting cargo such as Wnt5a and miR-155, which may enhance tumor cell invasiveness and resistance to anoikis, although TAM-exo-specific evidence for this mechanism is preliminary and requires confirmation in dedicated functional studies. MMP-2 and MMP-9 carried by TAM-exos have similarly been proposed to remodel the extracellular matrix and create metastatic tracks, drawing on the broader tumor-exosome ECM-remodeling literature (e.g., melanoma exosome effects on bone marrow progenitor cells; 58), though a TAM-exo-specific MMP-2/9 mechanistic study would strengthen this claim. Clinically, elevated plasma miR-21 and miR-155 have been associated with lymph node metastasis and reduced overall survival in colorectal, hepatocellular, and breast cancers in exosome-related contexts (59); whether this specific association is best attributed to TAM-exos, as opposed to exosomes from other tumor or stromal sources, requires direct verification. In a patient cohort study, exosomal PD-L1 from TAMs was associated with primary resistance to anti-PD-1 therapy in non-small cell lung cancer (18).Preclinical GW4869 treatment or Rab27a knockdown reduces TAM-exo secretion and partially restores anti-tumor immunity (27).

TAM-derived exosomes simultaneously silence cytotoxic immunity, recruit Tregs, remodel vasculature, and facilitate metastasis, making them a high-priority target for combination immunotherapy strategies. The heterogeneity of TAM-exo populations within the TME adds a further layer of biological complexity that is only beginning to be appreciated. Single-vesicle proteomics has revealed that M1-like and M2-like TAM subpopulations release exosomes with fundamentally distinct surface protein profiles: M1 TAM-exos are enriched in CD80, CD86, and HLA-DR, promoting DC maturation and CTL priming, whereas M2 TAM-exos display CD163, CD206, and IL-10, driving tolerogenic signaling in recipient immune cells (52, 55).It has been proposed that the M1-to-M2 TAM-exo ratio within a tumor correlates with patient prognosis across multiple solid tumor types, with a higher proportion of M2 TAM-exos associating with lymph node metastasis, vascular invasion, and reduced disease-free survival, by analogy with exosomal miRNA/PD-L1 findings reported in liver cancer macrophage models (59); a dedicated multi-tumor TAM-exo ratio study would be needed to confirm this specific prognostic correlation. This ratio is dynamically modulated by therapy: anti-PD-1 checkpoint blockade has been shown to shift the intratumoral TAM phenotype toward M1 in responding patients, concomitantly increasing the proportion of immunostimulatory M1 TAM-exos and reducing M2 TAM-exo-mediated suppression of CTLs (18). These findings suggest that TAM-exo profiling — both as a baseline prognostic biomarker and as an on-treatment pharmacodynamic readout — may provide clinically actionable information beyond what is currently captured by standard immunohistochemical TAM density scoring.

### 3.2 Mast cell-derived exosomes

In normal tissue and during acute infection, mast cells act as first-responder innate sentinels whose degranulation and exosome release recruit and activate other immune cells against pathogens; within the TME, tumor-derived prostaglandins, SCF, and CXCL12 co-opt this same recruitment machinery to draw mast cells into the tumor bed (53, 60). Once resident there, mast cell-derived exosomes (MCDEs) have been reported, primarily in preclinical models, to transmit cargo including IL-13, histamine, and PGE2 that drives Th2 immune deviation — typified by eosinophil recruitment, M2 macrophage polarization, and CD8+ T cell suppression (53); this specific exosomal cargo profile draws on the broader mast cell secretome literature and would benefit from dedicated MCDE proteomic confirmation. Exosomal miR-let-7a/b suppresses TLR4 signaling in DCs by targeting HMGA2 and LIN28B, impairing DC-mediated Th1 polarization and IFN-γ production *in vitro* (61). An analogous mast-cell-associated Th2 skewing has been linked to worse disease-specific survival in stage I lung adenocarcinoma in a clinical cohort (62); whether this prognostic association extends to colorectal, gastric, and pancreatic cancers, as has been proposed, requires direct confirmation in those tumor types. This TME-driven redirection of an otherwise protective sentinel function toward tumor-permissive signaling is a recurring theme across the myeloid populations considered in this section.

MCDEs have been reported to facilitate tumor vascularization through VEGF-A and Ang-1 delivery to endothelial cells (63); the specific activation of the VEGFR2/AKT/mTOR axis via this route draws on related exosome-driven DAMP/angiogenesis signaling literature and warrants MCDE-specific mechanistic confirmation. Bidirectional mast cell–tumor exosomal crosstalk has similarly been proposed to drive a reinforcing loop in which tumor-derived exosomes reprogram mast cells toward pro-tumorigenic secretory states while MCDEs promote tumor cell proliferation and survival signaling (AKT/ERK), by analogy with exosome-mediated proliferative signaling reported in other tumor-immune cell pairs (64); a dedicated mast-cell-tumor study would strengthen this specific claim. Microparticle/exosome-mediated transfer of P-glycoprotein and other multidrug-resistance-associated membrane proteins has been demonstrated between cells in other tumor contexts (65); whether MCDEs specifically mediate an analogous drug-resistance transfer to recipient tumor cells has not yet been directly tested and remains an open question for future mast-cell-exosome studies. Disrupting the mast cell–tumor exosomal loop — combining mast cell stabilizers with exosome secretion inhibitors — has demonstrated additive tumor growth inhibition in preclinical pancreatic cancer models.

Through Th2 immune deviation, tumor angiogenesis, and a still-emerging role in chemoresistance, mast cell-derived exosomes occupy a distinct mechanistic niche among the myeloid populations discussed here, and their enrichment in candidate actionable cargo makes them a plausible combination-therapy target pending further validation. An emerging dimension of MCDE biology is their capacity to modulate the spatial organization of the TME. In preclinical models, MCDEs bearing surface CXCL8 and CCL2 act as chemoattractant vectors, recruiting monocytes and additional mast cells to the tumor core, thereby amplifying the pro-tumorigenic myeloid infiltrate (54).This spatial crosstalk between MCDEs and monocyte recruitment creates a feed-forward loop in which escalating mast cell density drives progressive Th2 skewing, angiogenesis, and immune exclusion of CD8+ T cells. Whether mast cell-derived exosomes are detectable in ascites or other effusion fluids in ovarian and other peritoneal malignancies, and whether their cargo correlates with mast cell density or chemotherapy resistance, has not to our knowledge been directly reported and represents an open question for future liquid-biopsy studies. If confirmed, MCDE cargo profiling — particularly exosomal IL-13, PGE2, and VEGF-A — could offer a minimally invasive biomarker strategy for assessing mast cell–tumor axis activation and guiding decisions regarding mast cell-targeting adjuvant therapies.

### 3.3 Neutrophil-derived exosomes

The final myeloid population considered here, the neutrophil, shows the same N1/N2 duality already noted for TAMs and mast cells, but with its own distinct exosomal cargo. Tumor-associated neutrophils (TANs) adopt either anti-tumor N1 or pro-tumor N2 phenotypes under opposing TME signals, with TGF-β and G-CSF driving N2 polarization (66, 67).It is the N2 pole of this spectrum that dominates the immunosuppressive literature: neutrophil-derived exosomes (NDEs) from N2 TANs carry elastase, cathepsin G, MMP-9, and miRNAs including miR-143 and miR-145 that facilitate tumor invasion and metastatic niche establishment (68). The role of NDE-delivered miR-223 is less straightforward: in non-small cell lung cancer, neutrophil-derived exosomal miR-223 has been reported to suppress NLRP3 signaling and inhibit tumor progression (69), whereas exosome-delivered miR-223 in gastric cancer, N2-neutrophil exosomal miR-223 targets PTEN to activate PI3K/AKT and promote progression (70) — a context-dependent duality that the field has not fully reconciled, and this manuscript follows that unresolved literature rather than asserting a single direction of effect. Neutrophil extracellular traps (NETs) generated by N2-polarized neutrophils have separately been shown to cleave E-cadherin, disrupt adherens junctions, and promote EMT (71); whether an analogous, exosome-intrinsic MMP-9/E-cadherin mechanism operates independently of NET formation has been proposed but requires direct experimental confirmation.

Neutrophil extracellular traps (NETs) formed by these same neutrophils deliver citrullinated histones (H3Cit) and MPO to tumor and stromal cells, activating TLR4/NF-κB and inducing pro-metastatic chemokines (IL-6, IL-8, CXCL5) that establish the pre-metastatic niche (72); whether NDEs specifically (as distinct from NETs) carry the same H3Cit/MPO cargo and produce the same chemokine induction has not yet been directly tested. *In vitro* and murine tumor models indicate that NDE-transferred Arg-1 depletes L-arginine in the TME, blocking TCR ζ-chain re-expression and suppressing T cell proliferation (73). In contrast, N1 neutrophil NDEs have been proposed to carry TNF-α and cytotoxic cargo that suppress tumor cell proliferation and promote apoptosis; direct evidence for NDE-specific activation of anti-tumor immune responses in syngeneic models is still limited, and the closest available primary data instead show that tumor-derived (rather than neutrophil-derived) extracellular vesicles carrying TNF-α can induce apoptosis in NK cells (74) — an immunosuppressive, not immunostimulatory, effect that underscores how context-dependent TNF-α-bearing vesicle biology can be. The ratio of pro-tumor to anti-tumor NDE populations in the circulation of cancer patients has been proposed as a composite biomarker of treatment response and neutrophil polarization state (75). Neutrophil membrane-camouflaged nanoparticles exploit TAN tumor tropism for superior drug delivery in glioblastoma and colon cancer models (76).

The clinical translation of NDE biology is being accelerated by advances in single extracellular vesicle analysis technologies. High-resolution nanoflow cytometry and proximity ligation assay-based EV profiling have enabled simultaneous quantification of neutrophil-specific surface markers (CD66b, CD15) and immunosuppressive cargo (Arg-1, MMP-9) on individual NDEs isolated from patient plasma, without the need for bulk ultracentrifugation (68).Building on the established mechanistic link between myeloid Arg-1 activity, TCR ζ-chain suppression, and impaired T cell proliferation (73), exploratory studies in non-small cell lung cancer have begun to examine whether pre-treatment circulating NDE Arg-1 levels might predict resistance to platinum doublet chemotherapy; this specific NDE-biomarker application remains preliminary and awaits validation in larger clinical cohorts. Separately, in colorectal cancer patients undergoing curative resection, perioperative surges in neutrophil activation and NET formation have been linked to early hepatic metastasis, implicating surgery-induced neutrophil activation as a potential driver of post-operative metastatic seeding (71); whether NDE-specific (as opposed to NET-specific) cargo release follows the same perioperative kinetics has not yet been directly tested. Together, these exploratory findings support further development of NDE-based liquid biopsy panels as companion diagnostics for guiding neutrophil-targeted adjuvant strategies, pending validation in larger prospective cohorts. The extent to which these neutrophil-exosome mechanisms translate from preclinical models into reproducible clinical utility remains an open question, but the underlying biology positions NDEs as both a candidate immune-evasion mediator and a versatile scaffold for next-generation cancer nanomedicine. The myeloid-lineage mechanisms discussed in this section are summarized in **Table 2**.

**Table 2:** Myeloid cell-derived exosome subtypes (TAM, mast cell, and neutrophil lineages): molecular cargo, tumor-promoting and immunosuppressive functions, therapeutic targets, and key evidence.

Myeloid cell source	Key cargo	Primary functions	Therapeutic target	Key references
TAM-derived (M2 TAMs)	IL-10, TGF-β1, miR-21, miR-155, VEGF-A, MMP-2/9, PD-L1, Wnt5a	CTL exhaustion; Treg expansion; M2 loop reinforcement; tumor angiogenesis; EMT; metastasis	GW4869; Rab27a knockdown; anti-PD-L1	52, 59
Mast cell-derived	IL-13, PGE2, histamine, VEGF-A, Ang-1, miR-let-7a/b	Th2 immune deviation; tumor angiogenesis via VEGFR2/AKT/mTOR; bidirectional mast cell–tumor crosstalk; contribution to chemoresistance	Cromolyn sodium; VEGFR2 inhibition; miR-let-7 antagomirs	53, 60
Neutrophil-derived (N2 TANs)	Elastase, cathepsin G, MMP-9, miR-143, miR-145, miR-223, H3Cit, MPO, Arg-1	EMT via E-cadherin cleavage; cancer stem cell activation; NET-mediated pre-metastatic niche; T cell L-arginine depletion	PAD4 inhibition; MMP-9 inhibitors; miR-223 antagomirs	66, 67

## 4 Exosomes: from “cellular dust” to key immune mediators

The traces of exosomes from obscurity to prominence represents one of the most remarkable transformations in biomedical research. Initially dismissed as mere cellular detritus or molecular waste products destined for elimination, exosomes were largely overlooked by mainstream science (77). However, this understanding shifted dramatically over the past two decades as pioneering researchers recognized that these submicron vesicles constitute far more than cellular garbage; they function as sophisticated vehicles of intercellular communication. This conceptual revolution has reframed our understanding of how cells orchestrate complex biological processes, particularly in immune regulation. What was once regarded as a disposal mechanism has emerged as a fundamental mode of information transfer, reshaping our comprehension of cellular crosstalk in both health and disease (78). Currently exosomes are recognized as pivotal mediators capable of reprogramming immune responses and determining the fate of immune encounters.

Exosomes are a subtype of small extracellular vesicles (sEVs) defined by their endosomal origin. They are formed through the inward budding of the limiting membrane of multivesicular bodies (MVBs) and are released upon MVB fusion with the plasma membrane. While these vesicles are typically in the 30–150 nm size range, size alone is insufficient as a defining criterion. Populations of EVs are heterogeneous, and conventional characterization requires an orthogonal approach. This typically includes the detection of proteins commonly enriched in exosomes (such as tetraspanins CD9, CD63, CD81, and cytosolic proteins ALIX and TSG101), alongside particle concentration and morphological assessment. It is crucial to note that the characteristic ‘cup-shaped’ morphology, commonly observed by conventional transmission electron microscopy, is an artifact of sample dehydration and does not represent the vesicles’ native structure. More accurate morphological characterization can be achieved via cryogenic electron microscopy (cryo-EM), which preserves the native, hydrated state of the sample (79). Their biogenesis follows a precisely orchestrated pathway: cells first form early endosomes through membrane invagination, which mature into multivesicular bodies (MVBs) containing intraluminal vesicles. Upon fusion of these MVBs with the plasma membrane, the intraluminal vesicles are released into the extracellular space and are then termed exosomes (**Figure 4**) (4). The characteristic cup-shaped morphology of exosomes, readily identifiable through transmission electron microscopy, reflects their endosomal origin and provides a distinctive structural signature (80, 81). These vesicles carry a remarkably diverse cargo reflective of their parent cells, including proteins, nucleic acids (mRNA, microRNA, DNA), lipids, and metabolites (82). Their surface has specific marker proteins including tetraspanins (CD9, CD63, CD81), ALIX, and TSG101, which serve as reliable indicators for identification and characterization (83). These markers are not merely structural decorations; they facilitate recognition by recipient cells and determine which cellular populations will internalize the exosome and respond to its cargo. The selective loading of these marker proteins reflects an active biological process, not random packaging, indicating that exosomes represent intentional communication vehicles rather than passive repositories (84). The extracellular vesicle structure is heterogeneous, comprising at least three major categories that must be carefully distinguished. Microvesicles (or ectosomes), ranging from 100 to 1000 nanometers, originate from direct budding of the plasma membrane in response to cellular activation or stress (85). Apoptotic bodies, substantially larger at 1000 to 5000 nanometers, are generated during programmed cell death through membrane blebbing and fragmentation (86). In contrast, exosomes originate from internal endosomal compartments, as detailed above, and possess distinct biophysical properties and molecular compositions compared to these other vesicle classes (87). While all three categories participate in cell-to-cell communication, their distinct biogenesis pathways, size ranges, and cargo compositions confer upon them unique immunological properties. Exosomes typically carry higher densities of specific tetraspanins and display distinct protein-to-lipid ratios compared to larger vesicles (88). Importantly, microvesicles often display procoagulant phosphatidylserine on their surface and function prominently in hemostasis, whereas exosomes specialize in transferring regulatory nucleic acids and immune-modulating proteins (89). While exosomes certainly function as mediators of intercellular communication, their role extends far beyond passive information transfer. Exosomes do not simply transmit messages between cells; they actively instruct immune cells to adopt particular functional states, differentiate into specific subsets, or alter their proliferative and migratory behaviors (90). For instance, exosomes derived from regulatory T cells impose immune tolerance on recipient macrophages and dendritic cells, whereas tumor-derived exosomes actively suppress anti-tumor immunity by transferring immunosuppressive molecules to infiltrating lymphocytes (91, 92). Similarly, when a dendritic cell becomes activated by pathogen-associated molecular patterns or inflammatory cytokines, this activation state is encoded into the exosome proteome and lipidome; these exosomes then transfer this “activation signal” to naive or regulatory T cells, fundamentally redirecting their differentiation trajectory (93). Tumor cells are understood to exploit this regulatory capacity by secreting exosomes loaded with inhibitory ligands (such as PD-L1) and suppressive microRNAs that collectively reprogram infiltrating T cells toward exhaustion. As such these are not passive communication events but rather active regulatory interventions that reshape immune regulation and signaling events (94). Moreover, such regulatory capacity explains why exosomes derived from different cellular sources produce such divergent and even opposing immunological consequences; bone marrow-derived mesenchymal stem cell exosomes suppress inflammation, while tumor-derived exosomes accelerate immune evasion (95).

**Figure 4:**
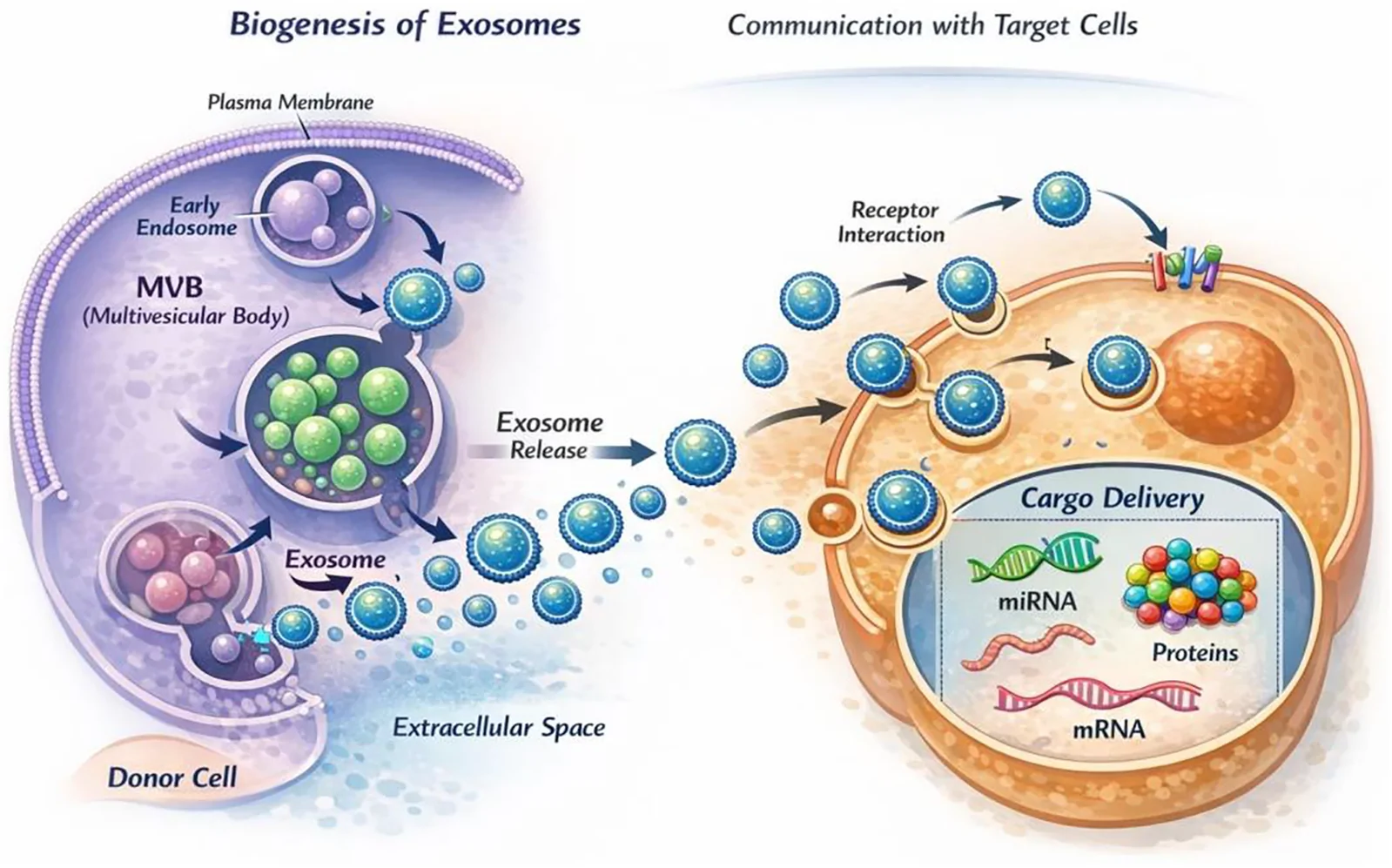
Biogenesis and intercellular communication of exosomes. Schematic representation of exosome formation and function. In donor cells, exosomes originate from early endosomes, which mature into multivesicular bodies (MVBs) containing intraluminal vesicles. Upon MVB fusion with the plasma membrane, exosomes are released into the extracellular space. These secreted exosomes subsequently interact with recipient cells through two primary mechanisms: direct receptor–ligand interaction at the cell surface or internalization leading to cargo delivery (including miRNAs, mRNAs, and proteins).

## 5 Mechanisms of exosome-mediated immune regulation

Exosome-mediated immune regulation operates through multiple integrated mechanisms that collectively enable cells to reprogram recipient immune populations at local and systemic scales (**Figure 5**). Direct receptor-ligand interactions initiate signaling cascades, while horizontal transfer of functional molecular cargo such as proteins, nucleic acids, and lipids allows donor cells to directly modify recipient cell phenotypes and functions. The particularly powerful mechanisms of cross-dressing enable dendritic cells to present an expanded antigenic repertoire without requiring processing, while death ligand-decorated exosomes induce immune cell apoptosis or exhaustion. Exosomal miRNAs mediate epigenetic reprogramming through altering chromatin states and histone modifications, and collectively, exosomal cytokine and chemokine cargo coordinate complex immune networks. Such multi-layered complexity demonstrates both the immunosuppressive effects exploited by tumors and the promising therapeutic potential of engineered exosomes for restoring anti-tumor immunity, treating inflammatory diseases, and enabling next-generation vaccination approaches.

**Figure 5:**
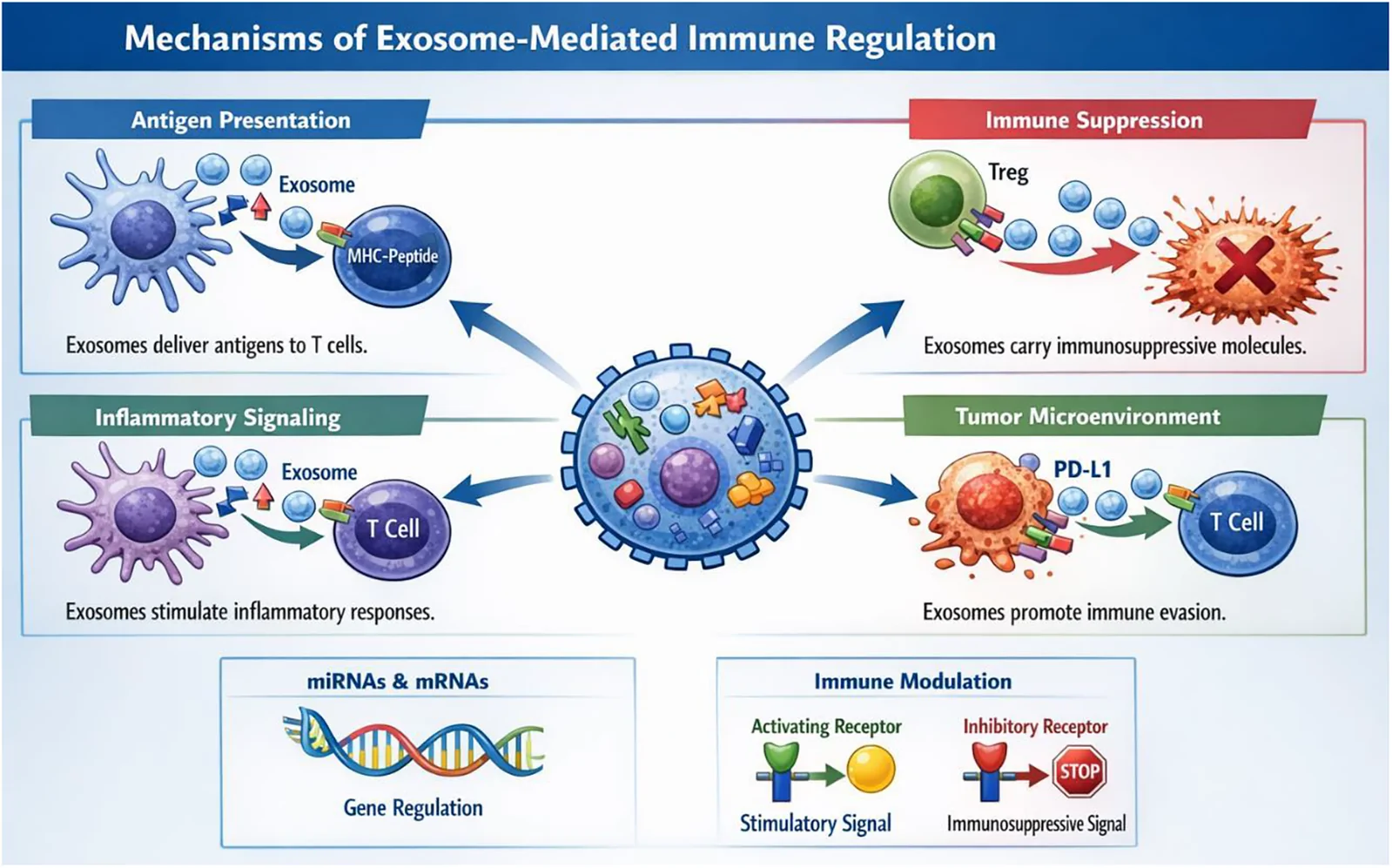
Mechanisms of exosome-mediated immune regulation. Exosomes act as key intercellular messengers in the immune system, capable of both activating and suppressing immune responses depending on their molecular cargo. They directly present antigens to T cells via MHC–peptide complexes, deliver pro-inflammatory signals to stimulate effector T cells, and carry immunosuppressive molecules that promote regulatory T cell (Treg) function. In the tumor microenvironment, exosomes expressing PD-L1 inhibit T cell activity, contributing to immune evasion. Additionally, exosomes shuttle microRNAs and mRNAs to recipient cells, enabling post-transcriptional gene regulation. The balance between stimulatory (activating receptors) and immunosuppressive signals ultimately dictates the outcome of immune modulation.

### 5.1 Direct receptor-ligand interactions on recipient immune cells

Exosomal surfaces display a sophisticated molecular architecture featuring tetraspanins, integrins, and immunologically active components that serve as primary mediators of receptor-ligand interactions with recipient immune cells. A mechanistic perspective on the immunological functions of exosomes has been proposed, highlighting that the molecular architecture of exosomes including ESCRT proteins, tetraspanins, RabGTPases, and lipid mediators directly contributes to immune function through precise mechanisms of exosome biogenesis and cellular targeting (96). It has been described that tetraspanins majorly function as molecular scaffolds that distribute proteins into highly organized microdomains consisting of adhesion, signaling, and adaptor proteins, organizing adhesion-mediated signaling through integrins and facilitating receptor clustering that amplifies downstream signaling cascade activation (97). Tetraspanin-enriched microdomains (TEMs) on exosomal surfaces coordinates immune cell recognition through their interaction with multiple ligands simultaneously, since tetraspanins has been identified among the most abundant proteins on extracellular vesicles that directly influence their uptake and subsequent immune cell activation through multivalent receptor engagement (98). The proteomic analysis of B cell-derived exosomes has revealed that they display abundant MHC class I and II molecules, heat shock cognate 70 and 90, and integrins including α4-integrin in tetraspanin-enriched lipid rafts typically resistant to detergent extraction, indicating organized functional complexes (99). These tetraspanin-enriched domains serve as sorting machines; as shown through high-throughput mass spectrometry these tetraspanin-enriched microdomain reveal clear pattern of interaction networks where proteins interacting with tetraspanin receptors present considerable degree of overlap, with highly specific CD81 tetraspanin ligands such as Rac GTPase being selectively routed to recipient cells, demonstrating selective cargo routing (100). Exosomal integrins engage with extracellular matrix components and specific receptors on immune cells, triggering integrin-dependent signaling that activates intracellular kinase cascades. It is understood that the both tetraspanins and integrins on exosomes are critical for their roles in cellular communication and function, with these molecules mediating precise biological effects through coordinated signaling (101). Moreover, recently integrin CD49d (α4 subunit of VLA-4) has been demonstrated to regulate immune synapse formation through actin rearrangements and synchronize downstream signaling through focal adhesion kinase (FAK)-actin axis interactions, demonstrating how integrin ligation initiates actin polymerization events fundamental to immune cell activation (102). Similarly, β1 integrin-induced ATP release through pannexin-1 hemichannels activates P2X4 purinergic receptors through a Pyk2-dependent mechanism, establishing an integrin-P2X4-ATP feedback loop that enhances T cell adhesion and migration (103). The specificity of these interactions is paramount since it has been demonstrated that CCL2 on exosomes specifically binds C-C motif chemokine receptor 2 (CCR2), triggering downstream signaling pathways that induce internalization of membrane integrin β1 and targeted lysosomal degradation, showing receptor selectivity in exosome-mediated immune modulation (104). Receptor-ligand interactions on exosomes culminate in activation of the mitogen-activated protein kinase (MAPK) cascade, particularly ERK1/2 phosphorylation. It is evident that the integrin β2 subunit CD18 functions as an immune recognition molecule that interacts with Syk kinase to activate extracellular regulated kinase (ERK), leading to H3K4 trimethylation and H3K9 acetylation, linking exosomal integrin engagement directly to epigenetic modifications in recipient immune cells (105). This represents a critical integration point where surface receptor ligation translates into chromatin remodeling.

### 5.2 Horizontal transfer of functional molecules (mRNA, miRNA, proteins)

The horizontal transfer of functional molecules via exosomes depends critically on selective packaging mechanisms that concentrate specific bioactive molecules into the vesicular lumen. For instance, the tumor-derived exosomes transfer oncogenic proteins, metabolites, and nucleic acids to both nearby and distant cells, inducing coordinated and complex molecular reprogramming in recipient cells through multifactorial cargo that synergistically activates multiple signaling networks (106). Lateral transfer analysis has revealed that extracellular vesicles transport drug resistance proteins and growth factors. Within each cancer hallmark signature, specific subgroups of these proteins are highly abundant and consistently expressed in exosomes, suggesting they are actively selected for inclusion rather than incorporated passively (107). Additionally, exosomal mRNA functions as authentic bioactive cargo capable of directing protein synthesis in recipient cells. It has been observed that mast cell-derived exosomes transfer functional mRNA between cells, resulting in expression of donor cell proteins in recipient mast cells, providing evidence that nucleic acids transferred via exosomes are not merely passive cargo but functionally active molecules capable of directing recipient cell protein synthesis (108). It also been explained that the mechanisms of action of extracellular miRNAs depend on their association with extracellular vesicles. MicroRNAs that are bound to extracellular vesicles can be taken up by cells and function like intracellular miRNAs (109). Exosomal miRNAs mediate post-transcriptional gene silencing through RISC complex assembly after delivery into recipient cell cytoplasm. Schwann cell-derived exosomes containing miRNAs modulate neurons, glia, endothelial, and immune cells through multifactorial cargo mechanisms, with studies establishing that Schwann cell-derived exosomes are powerful promoters of nerve repair capable of enhancing axon regrowth through miRNA-mediated effects (110). The functional impact is profound as it has been demonstrated that cancer-associated fibroblast-derived exosomes containing elevated microRNAs including miR-21, miR-378e, and miR-143 transfer these miRs to breast cancer cells where they reprogram stemness and epithelial-mesenchymal transition (EMT) phenotypes through repression of target genes such as p27Kip1 and induction of the PI3K/AKT pathway and aggressive state (111). The mesenchymal stromal cell-derived extracellular vesicles facilitate horizontal transfer of microRNAs, mRNAs, proteins, and lipids between cells through selective loading of functional molecules, with these cargoes modifying recipient immune cell phenotypes (112). Emerging evidence indicates that exosomal microRNAs (miRNAs) play a critical role in modulating inflammatory responses by directly targeting the mRNAs of key regulatory proteins. Specifically, exosomal miRNAs can suppress the expression of pro-inflammatory transcription factors while simultaneously promoting anti-inflammatory factor expression. Supporting this mechanism, Li et al. (113) demonstrated that exosomal miR-10b derived from protocatechuic acid-treated efferocytic macrophages inhibits endothelial inflammation by targeting the MAP3K7/β-TrCP/NF-κB signaling pathway, thereby suppressing a major pro-inflammatory transcription factor. In a complementary finding, Zhang et al. (114) showed that 3D hESC exosome-derived circular RNA hsa_circ_0076798 inhibits M1 macrophage inflammatory responses and alleviates liver fibrosis by regulating the miR-1184/DICER1 axis, which in turn suppresses the TNF/NF-κB pathway. A comprehensive proteomic characterization has shown that dendritic cell-derived exosomes contain annexin II, heat shock cognate hsc73, heteromeric G proteins (Gi2alpha), and MHC class I/II molecules along with milk fat globule-EGF-factor VIII, with the latter binding to integrins on dendritic cells and macrophages, suggesting specific targeting mechanisms (115]. Critically, one study has shown that when miRNAs modulate exosome release, they also affect the type of cargo delivered. Specifically, exosomes released from neurons under the influence of hsa-miR-22-3p carry pro-apoptotic cargo, which induces apoptosis in recipient glial and neuronal cells. This demonstrates that the miRNA content within exosomes directly determines the fate of recipient cells (116).

### 5.3 Antigen presentation via exosomal MHC-peptide complexes (cross-dressing of dendritic cells)

Dendritic cell cross-dressing involves acquisition of preformed peptide-MHC complexes through exosomal uptake and their direct presentation to T cells without requiring antigen processing. This process leads to dendritic cells acquiring preformed peptide-MHC class I/II complexes from cancer cells, either by taking up tumor-derived extracellular vesicles or through the horizontal transfer of plasma membrane fragments. Mechanistically, this acquisition depends on the recognition of phosphatidylserine. Specifically, it has been shown that dendritic cells rapidly obtain MHC I-containing membrane fragments from dead cells via phosphatidylserine-dependent pathways, and that this process is enhanced by the TLR3 ligand polyinosinic-polycytidylic acid (117, 118). The transfer of MHC-I molecules via exosomes relies on the recycling machinery already present in the recipient dendritic cells. Evidence from a study, using models of TAP dysfunction, shows that when both classical MHC-I presentation and cross-presentation dependent on the endosomal recycling compartment are impaired in dendritic cells, MHC-I molecules become trapped in the ER-Golgi intermediate compartment (ERGIC) (119). There, they are kept separate from Toll-like receptor regulation and instead are redirected into vesicular transport mediated by the ER-SNARE Sec22b. This allows them to meet with internalized antigen and restore cross-presentation, thereby revealing an unexpected flexibility in MHC-I trafficking pathways. The purinergic receptor P2X7 plays a key role in regulating the efficiency of cross-dressing. It has been observed that the P2X7 receptor (P2X7R) is essential for enabling cross-dressing between apoptotic cells and bone marrow-derived dendritic cells (BMDCs) (120). Notably, P2X7R must be present on both the apoptotic cells and the BMDCs to facilitate the transfer of membrane fragments and fully functional peptide/MHC complexes to the BMDCs. This ATP-sensing mechanism thus connects cellular stress signals to an increased capacity for antigen presentation. Further it has been suggested that the effectiveness of cross-dressed MHC-peptide complexes in T cell activation depends on their quantity and intracellular distribution. It was demonstrated using *in vivo* studies that MHC-I cross-dressing is crucial for anti-tumor CD8+ T cell priming by dendritic cells, where cancer cell MHC-I expression was required for optimal CD8+ T cell activation (121). Remarkably, dendritic cells were shown to acquire and display intact tumor-derived peptide: MHC-I molecules sufficient to induce antigen-specific CD8+ T cell priming without antigen processing. Exosomal proteins through proteomics analysis have shown enrichment in tissue-specific markers such as A33, cadherin-17, carcinoembryonic antigen, and epithelial cell adhesion molecules (EpCAM), suggesting that exosomal cargo includes structured antigen presentation molecules from their cellular origin (122). It is also understood that different dendritic cell subsets display distinct capacities for cross-dressing and exosomal MHC presentation. These dendritic-cell-derived exosomes possess functional MHC-I and MHC-II along with costimulatory molecules CD80 and CD86, facilitating antigen uptake, presentation, and activation of antigen-specific CD4 and CD8 T cells (123, 124). The quality and quantity of these molecules on DC exosomes directly determine their immunogenicity. It was observed that dendritic cells release exosomes together with phagocytosed pathogen, with bacteria in phagosomes surrounded by exosome marker-proteins suggesting formation of exosomes by inward budding into phagosomes, whereupon they are secreted together with phagosomal content, enabling selective loading of pathogenic antigens onto exosome-associated MHC molecules with implications for vaccine design (125).

### 5.4 Induction of apoptosis or exhaustion via death ligands (FasL, TRAIL, PD-L1)

Tumor-derived and immune cell-derived exosomes display death ligands such as Fas ligand (FasL), TNF-related apoptosis-inducing ligand (TRAIL), and programmed death ligand-1 (PD-L1) on their surfaces through selective packaging mechanisms. For instance, endometriotic tissue-derived exosomes carry the proapoptotic molecules FasL and TRAIL on their surface, functioning as decoys that induce apoptosis of activated peripheral blood mononuclear cells and Jurkat cells through these pathways, creating an immunosuppressive gradient protecting endometriotic lesions from cytotoxic attack (126). Exosomal PD-L1 engagement with T cell PD-1 receptors triggers inhibitory signaling that suppresses effector T cell function without requiring tumor cell-immune cell contact. It has been shown that colorectal cancer-derived exosomes transport PD-L1 into macrophages and impair CD4+ T cell function, with higher exosomal PD-L1 levels associated with more severe disease (127). It was also demonstrated using a nanoplasmonic cell-on-a-chip system that PD-L1+ exosomes from cancer cells significantly suppressed IFN-γ and IL-2 secretion in neighboring T cells, offering direct insights into exosome-mediated immune suppression through death ligand signaling (128). As such exosomal PD-L1 represents a distinct category of soluble immune checkpoint molecules with high prognostic significance for predicting immunotherapy response, as these vesicles shield tumor cells from immune recognition (13, 129). FasL on exosomes triggers death-inducing signaling complex (DISC) formation through Fas receptor clustering. This mechanism involves multivalent presentation of death ligands on exosomal surfaces enabling simultaneous engagement of multiple Fas receptors on target T cells, initiating caspase-8 and -10 activation followed by mitochondrial outer membrane permeabilization through Bid cleavage (130). TRAIL displayed on exosomes engages death receptors (DR4 and DR5) on T cells, activating both extrinsic apoptotic pathways through DISC formation and intrinsic pathways through mitochondrial dysfunction. A study has demonstrated that tumor-derived exosomal HMGB1 activates B cells and promotes the expansion of regulatory B cells (TIM-1+ Bregs) through the TLR2/4 and MAPK signaling pathways (131). This demonstrates that exosomal damage-associated molecular patterns (DAMPs) can influence immune cell phenotypes, steering them toward regulatory states. Furthermore, beyond direct death ligand signaling, exosomes modulate expression of inhibitory checkpoint molecules on immune cells. For instance, Liu et al. (132) show that Munc13-4 regulates PD-L1 sorting and secretion via exosomes to promote tumor immune evasion.

### 5.5 Modulation of cytokine and chemokine networks

Exosomes contain and deliver bioactive cytokines and chemokines that regulate immune cell recruitment, activation, and differentiation through paracrine signaling independent of direct cell-cell contact. The plasma exosomes from COVID-19 patients was found to carry SARS-CoV-2 double-stranded RNA (dsRNA) (133). When exposed to human peripheral blood mononuclear cells, these exosomes induce strong production of interleukin-6 (IL-6), IL-8, tumor necrosis factor-α (TNF-α), and other inflammatory cytokines and chemokines. This demonstrates that exosomal pathogen-associated molecular patterns (PAMPs) engage pattern recognition receptors (PRRs) to drive inflammatory responses. Exosomal cargo including heat shock proteins, nucleic acids, and HMGB1 activate toll-like receptors (TLRs) on endosomal compartments in recipient immune cells. As demonstrated septic mouse serum exosomes enriched with cytokines and chemokines play critical roles in T cell differentiation and chemotaxis, with exosomes from septic mice inducing differentiation of Th1/Th2 cells through specific delivery of IL-12 and IL-4 via antibody-targetable mechanisms (134). The downstream signaling cascades activated by these exosomal components include NF-κB and MAPK pathway activation. It has been reported that exosomes from chickens infected with H5N1 avian influenza virus carry viral proteins specifically nucleoprotein and non-structural protein into non-infected immune-related cells (135). Once transferred, these exosomal proteins drive the phosphorylation of ERK1/2 and p38, thereby activating the mitogen-activated protein kinase (MAPK) signaling pathway. This activation leads to the expression of pro-inflammatory cytokines, including IFN-γ, IL-1β, and CXCL8. Immune cells engage in complex communication networks with other immune components mediated by exosomes, cytokines, and chemokines, which are pivotal in shaping tumor progression and therapeutic response (136). The exosomes from mesenchymal stem cells modulate inflammation by reprogramming macrophages, while those from antigen-presenting cells promote synovial inflammation through cytokine signaling, revealing cell-type-specific roles for exosomal cytokine cargo in immune polarization (137). Exosomal chemokines create chemotactic gradients that guide immune cell movement and tissue entry. Evidence from a recent study shows that ethanol-activated microglial exosomes contain increased levels of MCP1 (monocyte chemoattractant protein 1). When these exosomes are delivered to recipient β-endorphin neurons, they elevate cellular MCP1 levels and activate CCR2-related signaling molecules, including inflammatory cytokines and apoptotic genes (138). These effects are blocked by a CCR2 antagonist, confirming that exosomal chemokines signal through chemokine receptor-dependent pathways. Exosomal IL-6 drives the IL-6 amplifier model by both stimulating IL-6 production in recipient cells and activating the STAT3 signaling pathway. As described earlier, IL-6 serves as a key amplifier in local immune activation (139, 140). Synergistic crosstalk between NF-κB and STAT3 leads to hyperactivation, which in turn triggers the production of multiple cytokines. As one of these target cytokines, IL-6 activates non-immune cells, establishing a positive feedback loop through the IL-6-STAT3 axis.

## 6 Conclusion

The journey of exosomes from dismissed cellular debris to recognized central players in tumor immunology exemplifies the transformative power of revisiting biological paradigms. This review has systematically dissected how exosomes derived from lymphoid and myeloid immune cells orchestrate a coordinated immunosuppressive network within the TME, disrupting the cancer immunity cycle at multiple critical junctures. Several key conclusions emerge from this synthesis.

First, immune cell-derived exosomes are not passive bystanders but active architects of tumor immune evasion. B lymphoma-derived exosomes deliver PD-L1, FasL, and immunosuppressive miRNAs that directly exhaust cytotoxic T lymphocytes while expanding Treg populations. T lymphocyte-derived exosomes exhibit subset-specific functions: Treg-exos carrying TGF-β1 and CTLA-4 suppress effector responses, whereas exhausted CTL-exos propagate bystander T cell dysfunction. NK cell-derived exosomes, though cytotoxic under physiological conditions, undergo TME-driven reprogramming that converts them into vehicles of NK cell dysfunction via miR-23a-mediated LAMP1 silencing. Second, the myeloid compartment — TAMs, mast cells, and neutrophils — extends immunosuppression through exosomal delivery of IL-10, TGF-β1, Arg-1, and matrix-remodeling enzymes that simultaneously silence immunity and facilitate metastasis. Critically, these myeloid-lineage exosomes engage in bidirectional crosstalk that creates self-amplifying immunosuppressive loops, suggesting that single-target therapeutic approaches may prove insufficient.

Third, the mechanistic diversity of exosome-mediated immune regulation — encompassing direct receptor-ligand interactions, horizontal transfer of functional nucleic acids, cross-dressing of dendritic cells with preformed MHC-peptide complexes, and modulation of cytokine/chemokine networks — underscores the redundancy and resilience of this intercellular communication system. The selective packaging machinery (hnRNPA2B1, YBX1, nSMase2) that concentrates immunosuppressive cargo into exosomes represents a vulnerability that can be therapeutically exploited.

From a translational perspective, exosomes offer a dual opportunity: as non-invasive liquid biopsy biomarkers and as therapeutic platforms. Circulating exosomal PD-L1, miR-21, and miR-155 levels correlate with disease stage, treatment resistance, and inferior survival across multiple malignancies, enabling real-time monitoring without repetitive tissue biopsies. Therapeutically, pharmacological inhibition of exosome biogenesis (GW4869, Rab27a knockdown) has demonstrated preclinical efficacy in restoring anti-tumor immunity, particularly when combined with immune checkpoint blockade. Furthermore, the engineering of exosomes with immunostimulatory cargo — co-stimulatory ligands, tumor antigens, or antagonistic miRNAs — represents a compelling cell-free immunotherapy strategy that circumvents the toxicity and manufacturing challenges associated with cell-based therapies.

However, critical knowledge gaps remain. The heterogeneity of exosomal subpopulations within the TME, the dynamic plasticity of exosomal cargo under therapeutic pressure, and the optimal strategies for selective inhibition of pathogenic exosomes without disrupting physiological exosome-mediated immune homeostasis require urgent investigation. Future research must prioritize single-vesicle analytical technologies to deconvolute exosomal heterogeneity, longitudinal studies mapping exosomal cargo evolution during immunotherapy, and rationally designed combination trials pairing exosome-targeted agents with established checkpoint inhibitors.
